# Sequence processing with quantum-inspired tensor networks

**DOI:** 10.1038/s41598-024-84295-2

**Published:** 2025-02-28

**Authors:** Carys Harvey, Richie Yeung, Konstantinos Meichanetzidis

**Affiliations:** 1 Quantinuum, 17 Beaumont Street, Oxford, UK; 2 Quantum Engineering Centre for Doctoral Training, University of Bristol, Bristol, UK; 3 Department of Computer Science, University of Oxford, Oxford, OX1 3QD UK

**Keywords:** Applied physics, Qubits, Computer science

## Abstract

We introduce efficient tensor network models for sequence processing motivated by correspondence to probabilistic graphical models, interpretability and resource compression. Inductive bias is introduced via network architecture as motivated by correlation and compositional structure in the data. We create expressive networks utilising tensors that are both complex and unitary. As such they may be represented by parameterised quantum circuits and describe physical processes. The relevant inductive biases result in networks with logarithmic treewidth which is paramount for avoiding trainability issues in these spaces. For the same reason, they are also efficiently contractable or ‘quantum-inspired’. We demonstrate experimental results for the task of binary classification of bioinformatics and natural language, characterised by long-range correlations and often equipped with syntactic information. This work provides a scalable route for experimentation on the role of tensor structure and syntactic priors in NLP. Since these models map operationally to the qubits of a quantum processor, unbiased sampling equates to taking measurements on the quantum state encoding the learnt probability distribution. We demonstrate implementation on Quantinuum’s H2-1 trapped-ion quantum processor, showing the potential of near-term quantum devices.

## Introduction

In recent years data has become a plentiful resource that has contributed to monumental advances in artificial intelligence (AI). Application of large language models which are trained on TBs of data, such as GPT-3^1,2^, GPT-4^3^, and LAMDA^4^, have entered the public sphere and have been met with justifiable awe. However, critiques of this unstructured approach remain prominent and the large amounts of redundancy in these energy-hungry models further encourage us to ask if, despite impressive results, alternative routes are worth exploring. It is believed that naturally occurring learning systems utilise priors, or biases, which provide scaffolding to neural wiring^5–10^. A motivating question regards the brain’s ability to generalise from sparse examples when artificial neural networks typically require such large amounts of data. *Compositional* generalisation argues that the presence of innate wiring rules predisposes the brain with a model for reasoning and concept creation. Such ideas paved the way for the development of *structured* learning models. The contemporary example is graph neural networks (GNNs)^11,12^ which achieve state-of-the-art performance with improved sample complexity^11–16^.

Tensor networks provide low-dimensional representations of high-dimensional data. They are a natural framework for learning as they are equivalent to probabilistic graphical models^26^, where we simply replace factorisations of probability distributions into marginals with factorisations of high-rank tensors into lower-rank tensors. They have created some recent excitement in the machine learning community as they can address some of the fields key criticisms including large redundancy, lack of *interpretability*^17–23^ and lack of rigorous theoretical results e.g. in comparing expressive power of different architectures^24,25^. Tensor networks also naturally describe quantum processes^35^. As such there has been excitement to find ways to use quantum spaces as feature space^36^, which in principle may lead to superpolynomial advantage for machine learning^37,38^. Such *quantum tensor networks*^39^ have valid operational interpretations as quantum computations, for example realised with parameterised quantum circuits (PQCs)^40,41^. A tensor network is said to be efficiently contractible when the cost of contraction is a polynomial in the bond dimension and network size. A more rigorous definition can be given in terms of tree width and in^57^ this is connected directly to the ability to simulate the corresponding quantum circuit. Since all our tensor networks have bounded tree width we refer to them as quantum-inspired models since we do not require a quantum device for execution. While it’s still true that for large enough bond dimension classical simulation would not be efficient, even in the case of low tree width, it is likely that training such a model would also not be feasible. As will be discussed in further detail, even in the case of efficiently contractable networks there is however scope for polynomial speed-up in sampling the probability distribution over a tensor network on a quantum device.

The data we study in the work is bioinformatics and natural language, but these models may be applied to any sequence data. The task of sequence modelling in general reduces to learning a probability distribution over symbols. Tensor networks natively fuse the successful *distributional* approaches for encoding meaning, or ‘semantics’^27,28^, with the *compositional* structures rooted in the field of theoretical linguistics^29^, or ‘syntax’. In the DisCoCat framework^30^, the first to introduce such an approach to modelling the semantics of sentences, word embeddings take the form of tensors which are composed according to the syntactic structure of the sentence in which they participate, resulting in a syntax-aware tensor network. Specifically, and in contrast to our work, the compositional schemes used follow parses of typeological grammars, such as pregroup grammar^31,32^, Lambek-calculus^33^, or context-free grammar^34^. The syntactic frameworks we utilise do not require post-selection to be a part of our models as is required for scalability with real quantum devices. An additional difference is that we allow syntactic operations to be trainable rather than have only the semantics embeddings be parameterised. Beyond the shared naturality of tensor networks in both language modelling and quantum, previous work has been motivated by possible polynomial quantum speedup^42^ and the relevance of contextuality where for example in^43,44^ minimal contextual extensions of Bayesian language models show advantage even for this ‘classical’ data. Quantum DisCoCat models have been constructed for syntax-aware quantum machine learning tasks^45^, as has been demonstrated in small-scale proof-of-concept experiments^46,47^.

There has already been some success using one-dimensional tensor networks for probabilistic sequence modelling^25,48,49^. However, from a physical point of view, the presence of long-range correlations (known as Zipf’s law) in language and biological data^50–55^ motivates the use of tree-like or hierarchical tensor network architectures^56–58^. Key examples of hierarchical tensor networks are the tree tensor network (TTN)^59^ and multi-scale entanglement renormalisation ansatz (MERA)^60^, initially introduced to capture power-law correlations in critical quantum many-body systems. In direct analogue to condensed matter physics we leverage the flexibility in architecture design of tensor networks to restrict the exponentially large feature space according to the correlation structure relevant to a given problem^61^. That is, we introduce an inductive bias on the space of models according to any known structure in the data and learning task at hand. Finally, noteworthy explorations of syntax-aware neural-based models include the recursive neural network of Ref.^62^, defined for the task of sentiment analysis, where the authors motivate the use of syntactic structure from the point of view of interpretability and explainability. Reference^63^ introduces syntax-aware neural-based models where even more linguistic information from a combinatory categorial grammar (CCG)^64^ is used, a grammar on which we will base our syntax-aware models in this work. Additionally, improvement in inference and machine translation tasks have been found using syntax-aware neural models in^65,66^.

In this work, we present a rich family of learning models in the common language of tensor networks. Another main contributions of this work is the introduction of methods for *efficient* implementation of large-scale quantum tree tensor network models. Hence this work provides tools for testing the benefits of including strong inductive biases, including syntactic structure, in tensor-based models for NLP. Additionally and as a first at this scale, we run several of our trained models on Quantinuum’s state-of-the-art ion-trap quantum computer showing its potential power even in the near-term.

## Models

**Fig. 1:**
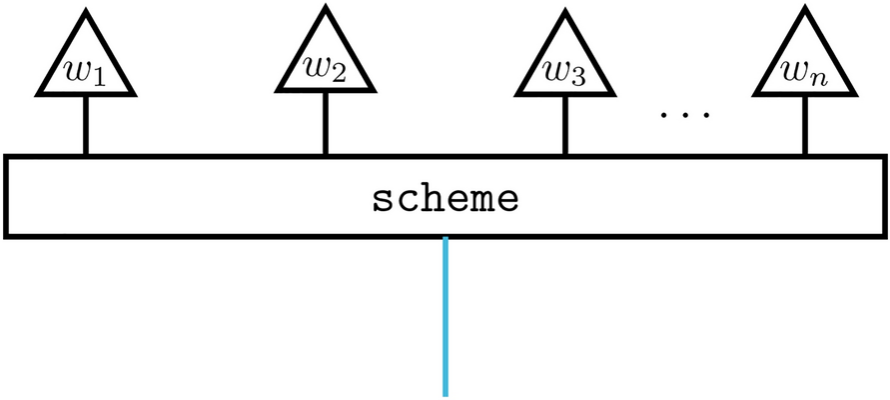
Process diagram representing a compositional scheme for a sentence. Black wires carry $\tau$ types and the blue wire carries the $\sigma$ type.

We introduce our models via a two-step process. To instantiate a model, first, a *compositional scheme* is defined for a sentence. In order to define a compositional scheme, we make use of the graphical language of process theories. The processes are depicted by *boxes*, with input and output *wires*. The compositional schemes we use throughout this work are such that wires carry one of two types, the ‘internal’ type $\tau$ and the ‘sentence’ type $\sigma$, as shown in Fig. 1. Freely composing the boxes, while respecting the types, allows for any process diagram to be generated, representing a scheme, to be defined. Given a vocabulary comprising a finite set of words, or more generally tokens, $V=\{w_i\}_i$, we consider compositional schemes for sentences, or more generally sequences, *S*, of finite length over this vocabulary.

Then, semantics is given via a *semantic functor*, *F*, i.e. a structure-preserving map. The semantic function maps the object of words to vectors and grammatical reductions to algebraic operations on these vectors. We may define *F* for arbitrary vector spaces. We will define *F* to give Hilbert space semantics to our compositional schemes, realising quantum tensor network models. This is done by assigning a number of qubits *q* to $\tau$ and $q'$-many bits to $\sigma$, and a parameterised quantum circuit (PQC) of a suitable size to each box, where the set of control parameters depends on the box:

- The *w*-box, which specifically prepares a *word-state*, typed $\tau ^{\otimes 0} \rightarrow \tau ^{\otimes 1}$, is mapped to a parameterised quantum state prepared by applying the circuit $U(\theta _w)$ on the fixed input state $|0\rangle ^{\otimes q}$. Note that to every word corresponds a parameter set.
- The *filter* *f*-box, with type $\tau ^{\otimes 2} \rightarrow \tau ^{\otimes 2}$, is mapped to $U(\theta _f)$ with 2*q* input and 2*q* output qubits.
- The *merge* *m*-box, typed $\tau ^{\otimes 2} \rightarrow \tau ^{\otimes 1}$, is mapped to a $U(\theta _m)$ with 2*q* input qubits and *q* output qubits, where the other *q* qubits have been either discarded or postselected by the $\bot$-effect.
- The *classifier* *c*, with type $\tau ^{\otimes 1} \rightarrow \sigma ^{\otimes 1}$, is mapped to a $U(\theta _c)$ which accepts a *q*-qubit state as input and outputs a $q'$-qubit state (by applying the $\bot$ effect) with $q' < q$ determined by the number of classes. This output is then measured in the *Z* basis to get a vector in $[0, 1]^{q'}$ which represents the probability of each outcome (as determined by the Born rule) and maps the output from quantum bits to classical bits.

**Fig. 2:**
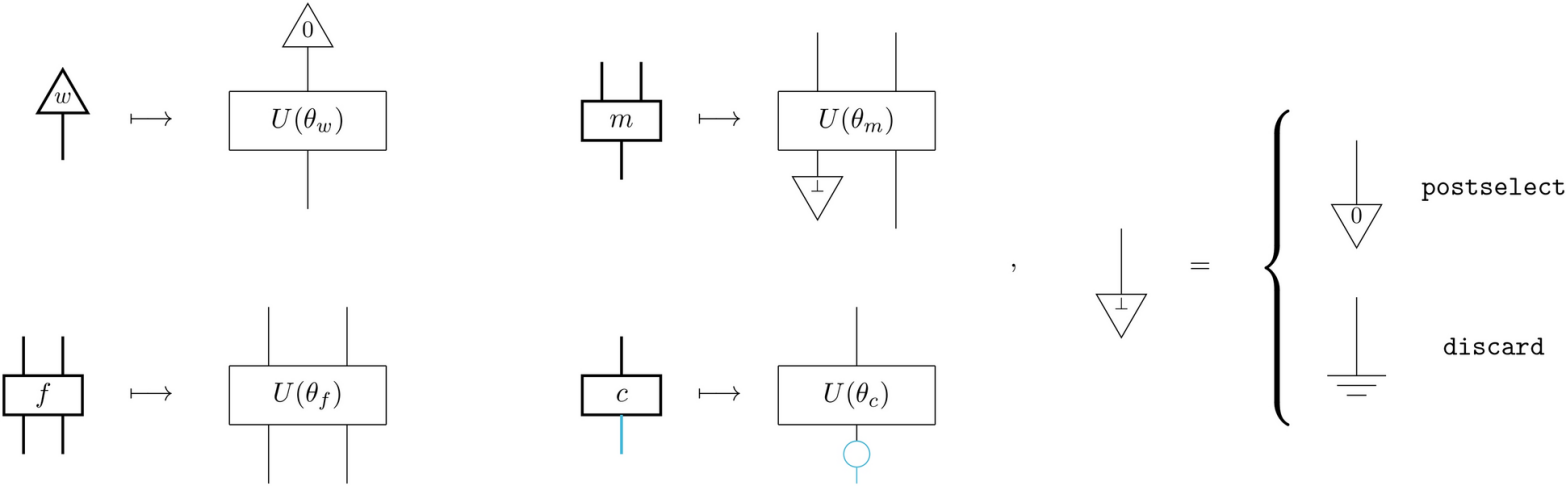
Definition of the semantic functor *F* which assigns parameterised quantum circuits $U(\theta _b)$ to boxes of type *b*. Thick black wires carry $\tau$ types and via *F* they are mapped to thin black wires carrying *q*-many qubits. The thick blue wire carries the $\sigma$ type and is mapped to a thin blue wire carrying *b*-many bits. For the $\bot$-effect, we can choose either to postselect on the all-zeros state or to discard.

Note $\tau ^{\otimes n}$ means *n* wires of type $\tau$ while for example $|0\rangle ^{\otimes q}$ denotes a *q* qubit state assigned to a wire after functor *F* is applied.The action of *F* on the scheme generators is shown in Fig. 2. In general, a more general class of quantum processes can be considered by introducing ancillae qubits to the parameterised quantum circuits.

For the $\bot$-effect, we consider two options. In the first case, $\bot =\texttt {postselect}$, it represents postselection, ie conditioning on the outcome of a nondeterministic measurement, by convention and without loss of generality to the all-zeros state. In the second case, $\bot =\texttt {discard}$, it represents discarding of that dimension, or a partial trace (marginalisation). The tensor network topology of the models we consider, inherited by the structure of the compositional schemes we define, allows for *efficient* tensor contraction strategies, thus making them classically simulable. This holds for both choices regarding the $\bot$-effect. We can therefore consider our models as ‘quantum-inspired’. At the same time, both versions have a valid operational interpretation as quantum circuits, so they could also be evaluated on quantum processors. However, while discarding is a *free* operation in quantum theory, postselection is not, as it incurs an *exponential* sampling overhead in number of qubits being postselected.

The number of qubits per wire *q*, the specific form, or ansatz, of each PQC realising a unitary *U*, and the choice for the $\bot$-effect are *hyperparameters* of our models. The ansätze we use are composed of a number of repeating layers of parameterised quantum gates. For simplicity, our functor is defined such that all PQCs involved are defined in terms of the same ansatz $U(\theta _b)$ and have the same number of layers *D*; the type of box *b* affects the control parameters $\theta _b$ as well as the size of the circuit. In the following, we taxonomise our tensor network models first by the compositional scheme defining their architecture, and then by the choice of semantic functor $F(\theta )$ and its hyperparameters, where $\theta = \cup _b \theta _b$ is the set of all control parameters (in the following we will suppress notation for *F* as it should be inferable by context). Rules and constraints we impose on the parameters that control PQCs define a model *species*.

### Path

**Fig. 3:**
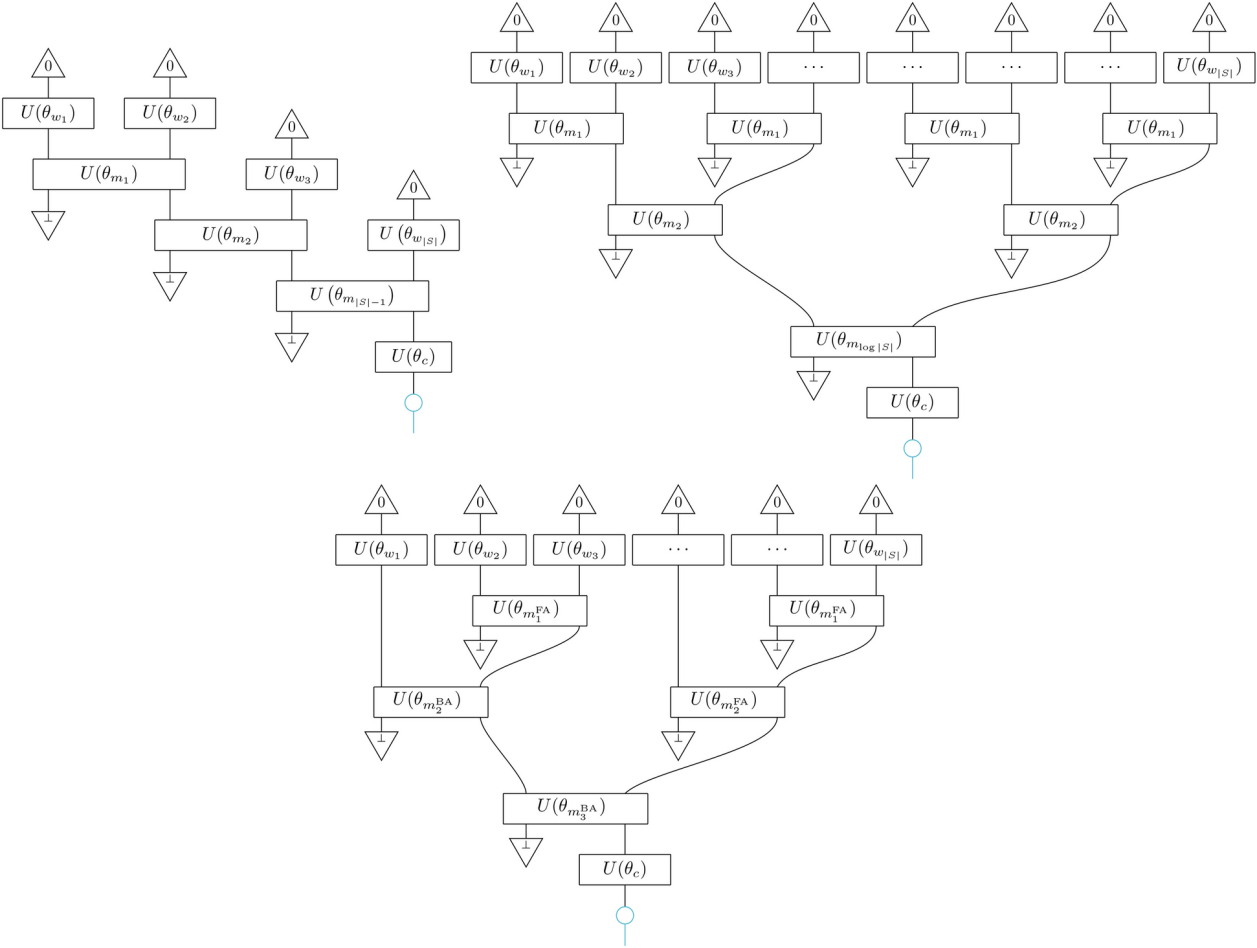
The scalable models: Path tensor network $F(\theta )[\texttt {path}]=\textrm{PTN}$ (top-left), tree tensor network $F(\theta )[\texttt {tree}]=\textrm{TTN}$ (top-right), and syntactic tensor network $F(\theta )[\texttt {syntax}]=\textrm{STN}$ for a given syntactic structure (bottom).

The first compositional scheme we study follows a sequential architecture which follows the *reading order* of the words in the sentence. Applying the semantic functor *F* defined in Fig. 2 to this scheme, we obtain the path tensor network model $F(\theta )[\texttt {path}] =\textrm{PTN}$, as shown in Fig. 3.

We denote the length of the input sequence *S* by |*S*|. If we allow a ‘hierarchical’ dependence of the parameter sets $\theta _{m_i}$ on their position $i\in \{1,2,\ldots ,|S|-1\}$, then we realise the hPTN models. Then imposing that all *m*-circuits share one parameter set $\theta _m=\theta _{m_i}, \forall i$, which makes the model recurrent, we realise the ‘uniform’ models uPTN. The uPTN can be interpreted as a vanilla version of a recurrent quantum model^67^, or a matrix product state (MPS) model where all the ‘physical dimensions’ are either discarded or postselected, save the last one which encodes the meaning of the sentence.

This compositional scheme does not take syntax nor long-range correlations into account and can thus serve as a baseline model against which syntax-aware models can be compared, but still satisfies the property that it is constructed by local application of a composition rule. Also, as it is effectively an MPS, it is not expected to be able to capture long-range correlations for a fixed bond dimension, constituting it a useful baseline against other models we construct below.

### Tree

The simplest model that is able to capture long-range correlations uses a compositional scheme with the topology of a *balanced binary tree*. We call this scheme tree. At every branching, an *m*-box combines two input wires into one. The number of branchings, and so the number of *m*-boxes is $|S|-1 = \sum _{i=0}^{{\log (|S|)-1}}2^i$, where the log is in base 2 throughout. This scheme has depth $\log (|S|)$, since at the *i*-th layer there are half as many parallel *m*-boxes than that in layer $i+1$. Figure 3 shows the result of applying the semantic functor to tree in order to obtain a tree tensor network (TTN) model.

We explore the ‘hierarchical’ species, hTTN, where circuits that belong to the *i*-th layer share a parameter set $\theta _{m_i}$. Furthermore, we define the ‘uniform’ species, uTTN, where all *m*-circuits share one parameter set $\theta _m=\theta _{m_i},\forall i$. The general form of quantum TTN is what is defined in Ref.^41^, where the authors allowed the *m*-circuits to have different parameter sets and used this model to classify images of handwritten digits (MNIST).

### Syntactic

We now present the first model that is syntax-aware, which we call the syntax. A CCG parser returns a syntax tree over text input which can be used directly as our compositional scheme. Structure-wise, it can be viewed as an intermediate scheme between path and tree, in the sense that the CCG parser may output binary trees that include path (right-branching or maximally-imbalanced binary tree) and tree (ie balanced binary tree), with path the limit case of maximal depth and minimal width and tree the limit case of minimal depth and maximal width. The number of *m*-boxes in this scheme is also $|S|-1$, but the depth depends on the syntactic structure. In Fig. 3 we provide an example of an instantiation of a syntactic tensor network (STN).

More specifically, we define three species of STNs. The $m_i^r$-circuits depend on the layer $i\in \{1,2,\dots ,|S|-1\}$, ie their distance from the leaves, as well as the CCG rule $r\in \textrm{R}$ as annotated by the parser. Similarly to the case of models based on the tree scheme, we can also, in this case, define a hierarchical species (hSTN), such that the *m*-circuits that are at depth *i*, where depth is defined by their distance from the leaves, share a parameter set $\theta _{m_i}=\theta _{m_i^r}, \forall r$. Also, since we have syntactic information, we can define a ‘rule-based’ species (rSTN), where the *m*-circuits annotated with the same CCG rule *r* share the same parameter set $\theta _{m^r} = \theta _{m_i^r}, \forall i$. Finally, we define the uniform species (uSTN), where all *m*-circuits share a single parameter set $\theta _{m}=\theta _{m_i^r}, \forall i,r$.

### Convolutional

**Fig. 4:**
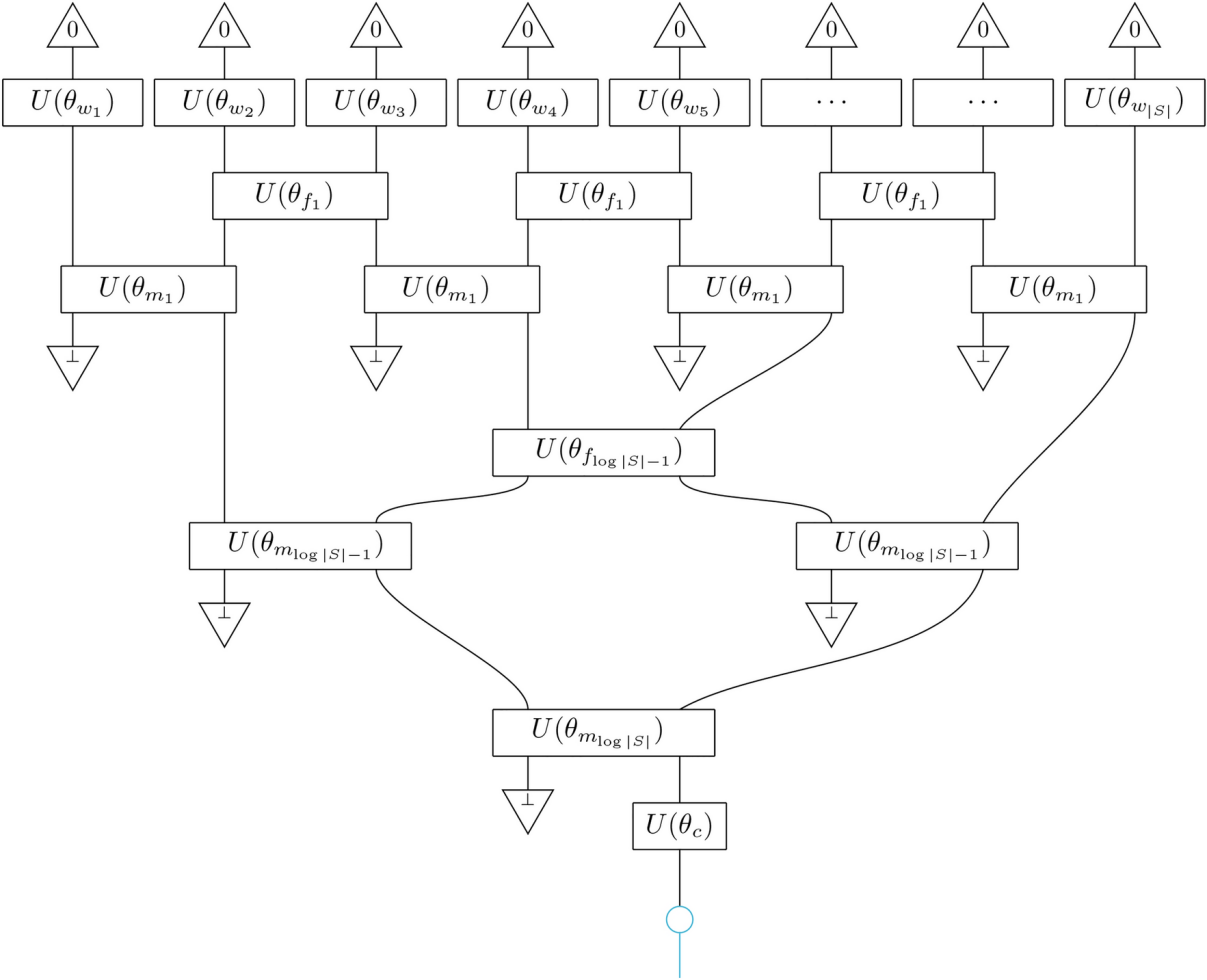
Convolutional tensor network $F(\theta )[\texttt{conv}]=\textrm{CTN}$.

We now introduce a compositional scheme that can be viewed as an enhancement of the tree, which we call conv. It is constructed by adding layers of *f*-boxes to the tree scheme, such that they act before the *m*-boxes on neighbouring wires that are not input to the same *m*-box. The number of *m*-boxes is $|S|-1$ and the depth is $\log (|S|)$ as is the case for tree. The number of *f*-boxes is $|S|-(\log (|S|)+1) = \sum _{i=0}^{\log (|S|)-1}(2^i-1)$, since at every layer there is one less *f*-box than there are *m*-boxes. The application of the semantic functor to this scheme in order to obtain *convolutional* tensor network $F[\texttt {conv}] = \textrm{CTN}$ is shown in Fig. 4. At the *i*-th layer, the layer of *f*-circuits filter out unnecessary entanglement and then the layer of *m*-circuits reduces the number of qubit wires, effectively coarse-graining the sentence and retaining only the relevant information for the task.

We define the ‘hierarchical’ models hCTN by having their parameter sets, $\theta _{f_i}, \theta _{m_i}$, be shared if they belong to the same layer. Finally, by making them be shared throughout the model, $\theta _f = \theta _{f_i}, \theta _m = \theta _{m_i}, \forall i$, we realise the self-similar ‘uniform’ uCTN. The CTN with $\bot = \texttt{discard}$ has been introduced in Refs.^68,69^ and implemented on quantum computers in Ref.^70^. In Ref.^68^ the authors put no constraints on the parameter sets $\theta _f, \theta _m$ and the model was used to classify MNIST, and in Ref.^69^ the authors introduce a special case of hCTN, where measurement-and-feedforward is used to keep the state pure. Finally, we define a ‘sliding’ variant which allows us to apply the convolutional model to large sequences efficiently. We call this the CTNS which consists of a sliding window of fixed size *w* that scans along the sequence. At each position in the sequence, *i*, a CTN is applied to the sub-sequence $[w_i,...,w_{i+w}]$. The outputs obtained from all sub-sequences are then aggregated by a function of our choosing, and here we choose the average.

### Syntactic convolutional

**Fig. 5:**
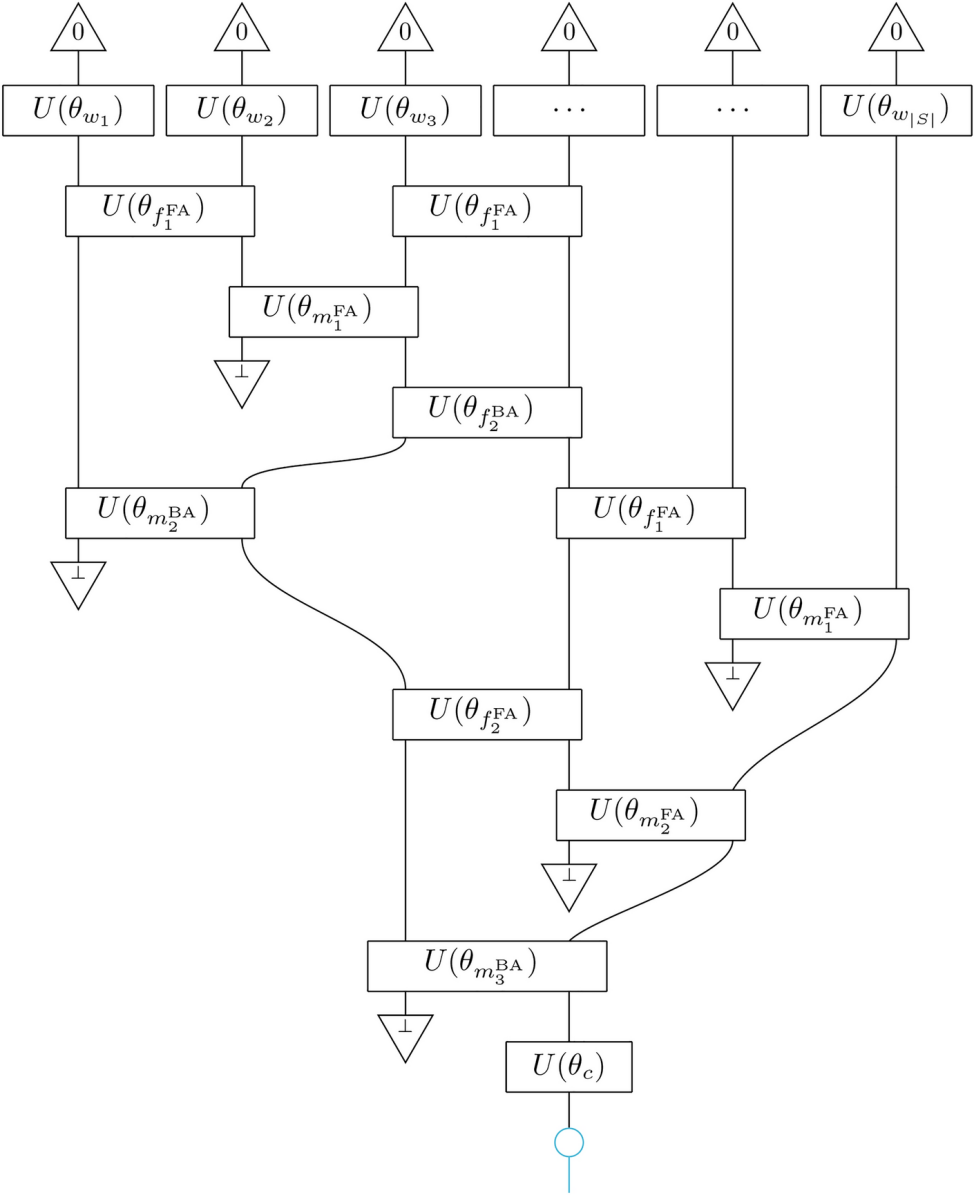
Syntactic convolutional tensor network $F(\theta )[\texttt {syntaxconv}]=\textrm{SCTN}$, for a given syntactic structure.

Finally, we introduce a compositional scheme that combines the features of syntax and conv, i.e. it is a syntax-aware coarse-grainer of a sentence, which we call syntaxconv. It is constructed by endowing syntax with *f*-boxes, following the convention that before every *m*-box we place *f*-boxes. The rule for placing an *f*-box is that it does not act on two wires that are inputs to the same *m*-box. In general, *f*-boxes do not necessarily commute, so we follow the convention that if two *f*-boxes act on the same wire, then the one that involves words that appear earliest in the sentence is applied first. The application of the semantic functor which returns the syntactic convolutional tensor network, $F[\texttt {syntaxconv}] = \textrm{SCTN}$, is shown in Fig. 5.

The *m*-circuits and *f*-circuits are controlled by parameter sets that depend on the CCG rule annotating the tree at each branching, as well as the depth (distance from the leaves), $\theta _{m_i^r}, \theta _{f_i^r}$. We define the ‘hierarchical’ models hSCTN, where circuits of the same depth share the same parameter sets $\theta _{m_i} = \theta _{m_i^r}$ and $\theta _{f_i} = \theta _{f_i^r}$, $\forall r$. We also define the ‘rule-based’ models rSCTN, in which case the parameter sets only depend on the CCG rule $\theta _{m^r} = \theta _{m_i^r}$ and $\theta _{f^r} = \theta _{f_i^r}$, $\forall i$. Finally, the uniform models uSCTN have two parameter sets shared across all *m*-circuits and *f*-circuits, $\theta _{m} = \theta _{m_i^r}$ and $\theta _{f} = \theta _{f_i^r}$, $\forall i,r$.

## Experimental results for binary classification

The models as we have defined them in section “Models” follow an ‘encoder’ architecture, in that they take words from a sentence as input, and return an output. Thus, they are designed to naturally act as *classifiers*. We focus on tasks of binary classification, ie only a single qubit ($q'=1$) is measured from the output quantum state of the $U_c$ circuit, resulting in the probabilities over two measurement outcomes, $p_0$ and $p_1$, as given by the Born rule. Each outcome is interpreted as the label of a class. Multiclass classification can be straightforwardly defined by setting $q' = \lceil \log L \rceil$ for a number of class labels *L*. Since all the model species we have introduced have a *tree-like* architecture, they can be evaluated *efficiently*; we provide the upper bounds to their contraction complexities in the Supplementary Material along with additional model details. Importantly, tree-like models are immune by construction to the phenomenon of barren plateaus during training^71–73^.

The models are defined using the DisCoPy library for monoidal categories^74,75^. Where applicable, the syntactic structures were obtained using the CCG parser Bobcat^76^, which is available in the lambeq package^77^. We simulate the models using the tensornetwork library^78^. Training is done using the library JAX^79^; by defining the forward pass as a pure JAX function, we are able to exploit Just-In-Time (JIT) compilation to batch compute the outputs of multiple sentences, even if the tensor networks were produced from schemes with different syntactic structures. While we experimented with different families of quantum ansätze, the results we present use the expressive ansatz 14 from^80^, as it had the best test performance. The parameters of a model are optimised using AdamW^81^ such that the labels of the train set $l_i\in \{0,1\}$ are predicted correctly according to the binary cross-entropy loss, $H = - \frac{1}{|\mathrm {train ~set}|} \sum _{i=1}^{|\mathrm {train ~set}|} l_i \log _2({p_1}_i) + (1-l_i) \log _2(1-{p_1}_i)$. If one were to train on a quantum computer, one would use the parameter-shift rule for estimating analytic gradients^82^, or use gradient-free optimisers such as SPSA^83^, which requires much less overhead and has been shown to perform well when training PQCs on near-term hardware^85^.

A validation set is used for model selection with early stopping. The model hyperparameters are the embedding qubit number and ansatz depth, (*q*, *D*), and learning rate. The optimiser is given a fixed random seed for all models. Finally, an unseen test set is used to evaluate the model’s generalisation performance as measured by the prediction accuracy. All test accuracy results are taken at best validation accuracy over the stated hyperpameters and learning rate with fixed seed rather than an averaged behaviour.

We work with three datasets comprising labelled sequences: two NLP datasets comprised of short news titles (Clickbait)^86^ and longer movie reviews (Rotten Tomatoes)^87^ and one bioinformatic dataset consisting of DNA sequences^88^. The classes are clickbait/non-clickbait, positive/negative sentiment and binding affinity respectively. The NLP data is lemmatised, which occurs post-parsing for the syntactic models. While PTN, TTN, STN are all efficiently batchable and scalable, CTN and SCTN remain harder to scale in simulation, as discussed in the Supplementary Material.

Table 1 show results only for the scalable models on the full datasets. Additionally, we provide a baseline LSTM model in a comparable parameters regime implemented using Keras^84^ and again using AdamW. We found that on the equivalent data the simple LSTM models considered consistently overfit showing significantly worse accuracy on validation and test set data than on the train set. This is especially true for the movie reviews which had a train accuracy of around 100 but a test set accuracy of random guessing.

**Table 1:** Test accuracies for all scalable models.

	Protein sequences	Titles	Reviews
Discard models			
uPTN	71.0 (1, 2)	98.2 (3, 1)	72.6 (1, 1)
hPTN	70.0 (1, 1)	98.7 (3, 1)	71.1 (1, 1)
uTTN	74.0 (1, 2)	98.6 (1, 2)	72.7 (1, 2)
hTTN	72.5 (1, 2)	98.0 (1, 2)	66.5 (1, 2)
uSTTN		98.9(1, 2)	69.8 (1, 2)
hSTTN		98.7 (1, 2)	67.8 (1, 2)
rSTTN		98.6 (1, 2)	70.7 (1, 2)
uCTNS	76.0 (1, 2) (4)		**76.76** (1, 2) (4)
hCTNS	**81.0** (1, 2) (8)		75.63 (1, 2) (4)
uCTN	72.5 (1, 1)		
hCTN	**80.0** (1, 2)		
Postselection models			
uPTN	72.5 (1, 1)	99.2 (1, 2)	74.3 (1, 1)
hPTN	70.0 (1, 1)	99.4 (1, 2)	68.0 (2, 1)
uTTN	74.0 (1, 2)	98.7 (1, 2)	70.7 (1, 1)
hTTN	80.5 (1, 1)	99.3 (1, 1)	67.1 (2, 2)
uSTTN		99.5(1, 1)	72.8 (1, 2)
hSTTN		99.2 (1, 2)	67.2 (1, 2)
rSTTN		99.4 (1, 1)	70.5 (1, 2)
uCTNS	79.0 (1, 2) (8)		75.16 (1, 1) (4)
hCTNS	80.0 (1, 2) (4)		74.51 (1, 2) (4)
uCTN	89.0 (1, 1)		
hCTN	**94.0** (1, 1)		
Baselines			
LSTM(3, 22)	71.5	71.1	52.5
LSTM(3, 51)	70.5	72.0	51.6
LSTM(3, 88)	70.0	73.0	51.1

For the tensor network models we state the best score over hyperparameters (*q*, *D*)(*w*). For Protein Sequences $q=1$ only and $q\in \{1,2,3\}$ otherwise. $D\in \{1,2\}$, $w\in \{4,8\}$. For the baselines, the number of parameters in the model are shown in brackets with the model name with number per word embedding followed by number in the remaining LSTM architecture. For the protein sequences the train/val/test split is 1600/200/200. For the news titles the train/val/test split is 25,312/3165/3165. For the movie reviews the train/val/test split is 8362/1046/1046.

Significant values are in [bold].

In order to obtain results from SCTN, we extracted a ‘reduced’ news titles dataset by implementing batching of sentences with the same syntactic structure and utilising vectorisation. These results are shown in Table 2 along with the CTN model for comparison and the restriction $q=1$ only.

**Table 2:** Test accuracies for SCTN and CTN model for the reduced dataset.

	Titles reduced
Discard models	
uCTN	** 96.0** (1, 2)
hCTN	95.0 (1, 1)
uSCTN	92.7 (1, 1)
hSCTN	86.0 (1, 1)
rSCTN	93.1 (1, 1)
Postselection models	
uCTN	** 97.0** (1, 1)
hCTN	94.9 (1, 2)
uSCTN	96.3 (1, 2)
hSCTN	82.8 (1, 1)
rSCTN	90.2 (1, 2)
Baselines	
LSTM(3, 22)	61.6
LSTM(3, 51)	57.8
LSTM(3, 88)	60.9

$q=1$ only and $D\in \{1,2\}$. The train/val/test split is reduced to 2033/763/763.

Significant values are in [bold].

We find that all our models train well in the low parameter regime. As is already known in NLP, it is not possible to show significant advantage for including syntax for sentiment analysis tasks. As shown in Table 1, for the simplest dataset consisting of news titles all models show good generalisation performance with improvement on test score over the LSTM.  For the case of the movie reiews, the CTNS performs the best but this is only significant in the case of discarding.  The most significant advatage of this model can be seen in the genetic data set with models restricted to $q=1$. For this dataset, the CTNS performs well but the complete CTN significantly outperforms all other models and the baselines. For the uCTN achieving **89.0**$\%$ test, the number of trainable parameters is 3/token and 11 in the remaining architecture. For the hCTN achieving **94.0**$\%$ test, the number of trainable parameters is 3/token and 8/layer in the remaining architecture (with 4 tokens and 6 layers in total) and again 3 for the final classification box. See Table 2 in Supplementary Material for exact parameter values for all cases. For the training with significantly reduced data shown in Table 2, all convolutional models significantly outperform the comparable LSTM with the uniform CTN scoring the best. The CTN model’s performance supports the importance of an architecture’s bias towards the correct correlations in achieving consistent resource compression even for relatively simple tasks. 

Finally, we ran a larger scale 50,000 review IMDb dataset using the uCTNS model with $\bot =\texttt {postselect}$, window size 4 and ansatz depth 1, achieving a test set accuracy of **88**$\%$. We did not do a full hyperparmeter grid search for this data but this is comparable to recent baseline results^89^ again with only 3 trainable parameters per word and 11 in the remaining architecture. Together these results support the potential of tensor networks for reducing resources in training models. In the future, experiments utilising the scalable machinery developed in this work should be applied to more complex tasks to test the robustness of these arguments.

### Execution on a trapped-ion quantum processor

**Fig. 6:**
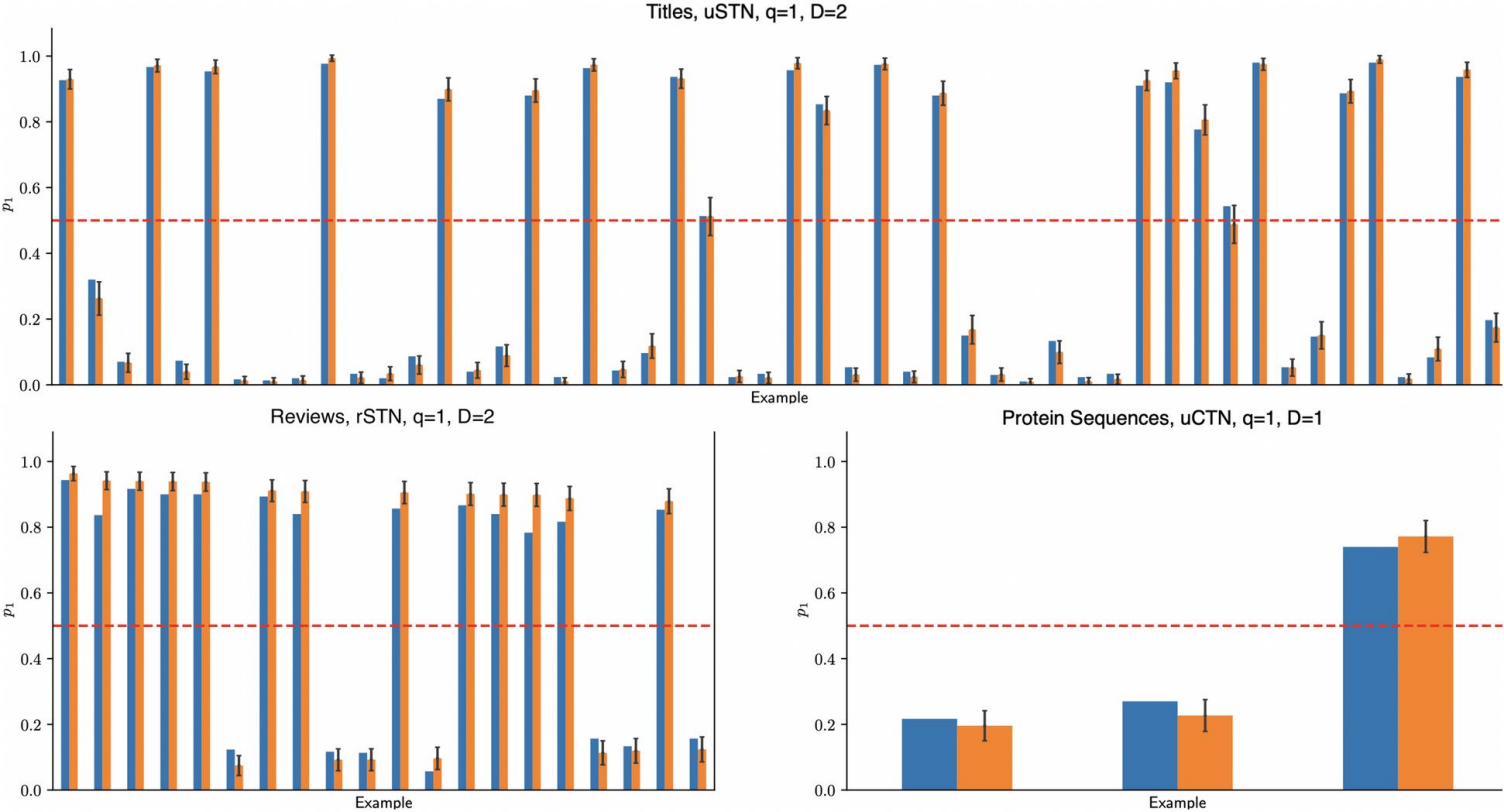
Test-time results for representative examples from the datasets used in this work for some choice of models. The sampled average $\tilde{M} = \frac{1}{300} \sum _{i=1}^{300} M_i$ (blue), obtained by execution on the H2-1 quantum processor for 300 shots, and the expected value $\mathbb {E}(M)$ (orange), obtained by exact classical noiseless simulation, of the measurement outcome *M*, which is a Bernoulli random variable. Specifically, $p_1 = \mathbb {P}(M = 1) = \mathbb {E}(M)$ represents the probability of measuring 1, and the red line represents the decision threshold (0.5). Error bars show the range of 2 standard deviations of *M* (95% confidence interval) away from the expected value $\mathbb {E}(M)$, considering only shot noise.

Finally, we execute a representative selection of test set examples from each dataset, utilising the uSTN, rSTN and uCTN models with $\bot = \texttt {discard}$, on Quantinuum’s 32-qubit H2-1 quantum processor with reported quantum volume of $2^{16}$^90^. The sampled averages over 300 shots are shown in Fig. 6 which are in good agreement with the simulations. For the shorter circuits relevant to classifying the new title dataset, we find that the estimated probability $p_1$ that determines the classification label agrees with its exact classical noiseless simulation, considering the variance arising from shot noise. We find that only 1 disagrees with its classical simulation regarding the classification label, due to both shot noise and the fact that for that circuit the exact result for $p_1$ is very close to the classification threshold. For the selected larger circuits corresponding to the movie reviews and protein sequences, we find that noise effects beyond shot noise, such as gate noise, have affected the estimated value of $p_1$. However, after considering the size of these circuits, it is notable that the classification label is not affected, since noise has not altered $p_1$ enough for it to cross the classification threshold. Before the quantum circuits corresponding to these models can be run on a specific backend, they need to be compiled, for which we used Quantinuum’s TKET compiler^91^. This entails translating the circuit’s gates into the native operations available to the device. The quality of the experimental results in relation to exact classical simulation is particularly impressive for the DNA binding sequences comprising 64 nucleotides each. In particular, the uCTN model, post-compilation, is a 64-qubit circuit that contains 1207 gates, of which 360 are two-qubit entangling gates, which are two orders of magnitude noisier than single-qubit gates. Furthermore, H2-1’s mid-circuit measurement and qubit-reset features allow for qubit reuse^92^. This allows us to reset a qubit that is no longer in use such that when we ‘discard’ the output in one part of our circuit we may use this qubit as a fresh input to a later operations. Remarkably, with qubit-reuse strategies, which essentially follow the logic of good tensor contraction strategies, the 64-qubit uCTN circuit is compressed down to an 11-qubit circuit.

## Discussion and outlook

We have introduced machine learning models for sequence classification constructed using the techniques of tensor networks. The architecture of the models plays the role of an inductive bias motivated by the inherent correlation and compositional structures present in the data. Moreover, we utilise unitary complex tensors which allows for their instantiation as parameterised quantum circuits. We have demonstrated both in simulation and by execution on a state-of-the-art trapped-ion quantum processor the efficient implementation of our models for datasets relevant to natural language processing as well as bioinformatics, showing good performance. These models show particular promise in generalisation power in the low parameter and small data regime. The models and methods defined in this work enable for the first time the experimentation with a variety of quantum-inspired tensor network models for sequence processing. Importantly, with the methods presented here, one can employ large-scale and real-world data, and explore the question of for what kinds of tasks and datasets is a syntax-aware structure beneficial, as well as explore more sophisticated model-selection strategies. To this end, we make our code and results available in this repository https://github.com/CQCL/classification-with-qttn.

In analogy to standard NLP techniques we can train quantum-inspired word embeddings and test their performance in a downstream task, such as the classification tasks tackled in this work. This can be done with the usual objectives such as the skipgram^93^ or glove^94^ methods, and it would be interesting to evaluate such embeddings in tasks such as word analogy. Creating embeddings in this way constitutes the creation of *quantum data* to be classified; interestingly, it is in this context in which quantum convolutional neural networks (CTN with discard in our work) were introduced^69^. In addition, our methods can be straightforwardly extended so that a syntactic structure is learned using adaptive methods applicable to our STN or SCTN models^95^, which could be used to infer syntactic structures in bioinformatics data^96^. Our models can also be applied to other highly-structured data similarly displaying long-range correlations, such as neuron firing networks^97^.

Beyond classification, our simple setup can be extended to accommodate generative modelling for sequences, using tree-like Born machines with a basis encoding of the vocabulary ($q=\log _2(|V|)$. In general, one can create a quantum language model using one of our non-syntactic models, which is trained in a masking task to capture the conditional probability distribution of tokens in text corpora. Additionally, there is scope for polynomial advantage in sampling from such models using a quantum device. To do this we simply have ancilla on the isometries, run the circuit with *O*(*log*|*S*|) gates and perform a measurement on the *q*|*S*| qubit output state to obtain a sample from the $2^{q|S|}$ possible strings. In simulation we need to contract the tensor network in order to obtain the probabilities over the possible strings where the complexity of contraction is *O*(*poly*(*log*|*S*|)). We note that for even for the sequences considered in this work we would need to resort to weak simulation to extract samples over a full *q*|*S*| qubit output sequence. Here, weak simulation refers to calculating the marginal probability distribution of a subsystem, updating the state of the subsystem with a sample (or *x* samples if following multiple probabilistic paths) and then extracting the marginal over the next subsystem, sampling and so on.

Finally, we consider two general directions, guided by the efficiency of contracting tensor network models. In this work we have only used the Born rule as a nonlinearity for obtaining the classification label, since we designed our models as to have valid quantum operational semantics. But one could use any element-wise nonlinearities one desires, acting on the complex-valued tensors^98^. Alternatively, it would be interesting to consider text-level syntactic structures^99^, which are conjectured to lead to hard-to-contract tensor networks. In that case, given quantum semantics, an efficient implementation would necessarily require quantum processors.

## Supplementary Information

Supplementary Information.

## Data Availability

All code and datasets required to recreate the results of this paper can be found in the repository https://github.com/CQCL/classification-with-qttn.
