# Supplementary material for: Sequence processing with quantum-inspired tensor networks
Source: Sci Rep. 2025 Feb 28;15:7155. doi: 10.1038/s41598-024-84295-2 (PMC11871337; doi:10.1038/s41598-024-84295-2)
Supplement: Supplementary file 1 — Supplementary Information. [file 41598_2024_84295_MOESM1_ESM.zip › main.pdf]

Supplementary Material: Resource-Efficient Sequence Processing with Quantum-Inspired Tensor Networks

Carys Harvey*[1,3], Richie Yeung[2,3], and Konstantinos Meichanetzidis[3]

[1]Quantum Engineering Centre for Doctoral Training, University of Bristol, BS8 1UD, UK
[2]Department of Computer Science, University of Oxford, OX1 3QD, UK
[3]Quantinuum, 17 Beaumont Street, Oxford, OX1 2NA, UK

July 27, 2024

1 Further model details

The compositional `schemes` used to instantiate the quantum tensor network models via the semantic functor F are shown in Figures 1-5 below. Black wires indicate τ types and blue wires indicate σ types. For every model species, we show the complexity of full tensor contraction in Table 1 and the total number of parameters in Table 2.

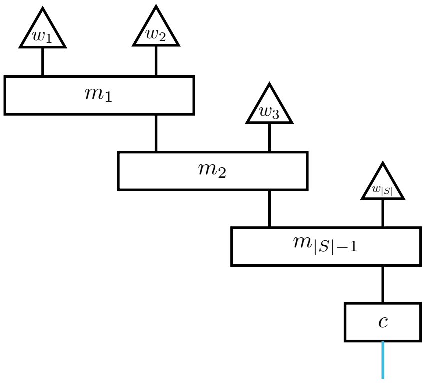

Figure 1: `path`.

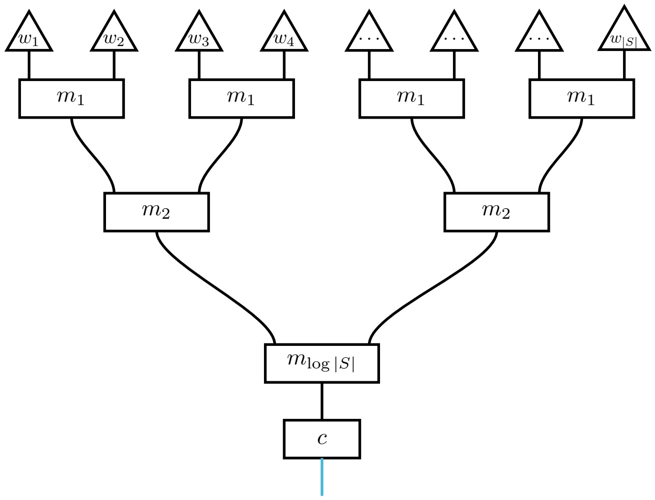

Figure 2: `tree`.

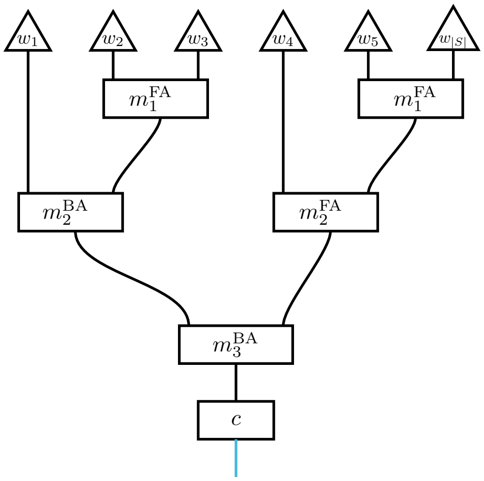

Figure 3: `syntax`, for a given CCG parse.

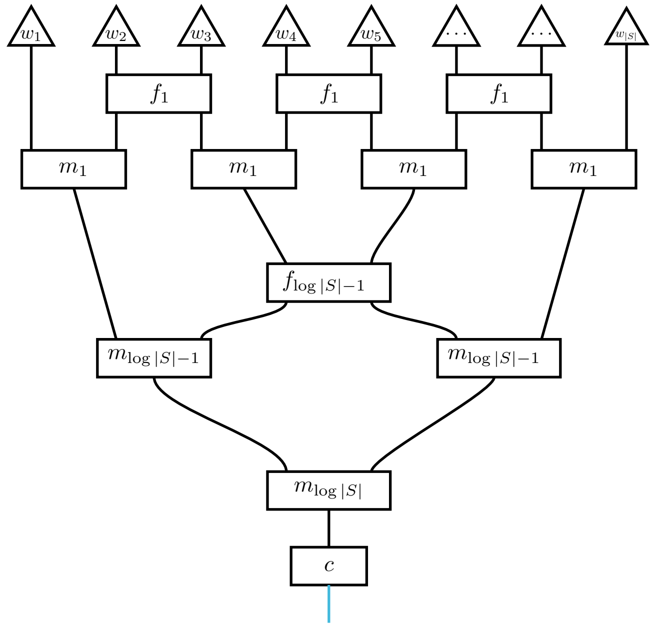

Figure 4: `conv`.

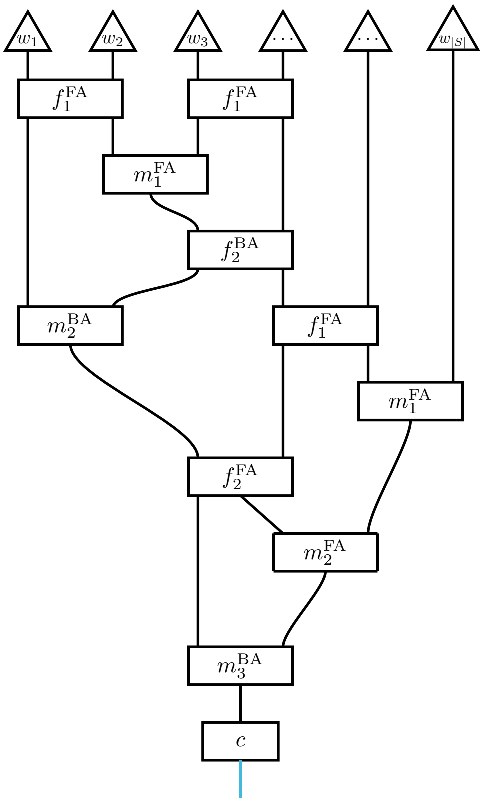

Figure 5: `syntaxconv`, for a given CCG parse.

$\perp$	PTN	TTN	STN	CTN	SCTN
`discard`	$\mathcal{O}(\lvert S\rvert\chi^4)$	$\mathcal{O}(\lvert S\rvert(\chi^2)^3)$	$\mathcal{O}(\lvert S\rvert(\chi^2)^3)$	$\mathcal{O}((\chi^2)^{2\log(\lvert S\rvert)})$	$\mathcal{O}((\chi^2)^{2\log(\lvert S\rvert)})$
`postselect`	$\mathcal{O}(\lvert S\rvert\chi^3)$	$\mathcal{O}(\lvert S\rvert\chi^3)$	$\mathcal{O}(\lvert S\rvert\chi^3)$	$\mathcal{O}(\chi^{2\log(\lvert S\rvert)})$	$\mathcal{O}(\chi^{2\log(\lvert S\rvert)})$

Table 1: Contraction complexity upper bounds for quantum and classical models as a function of the wire dimension $\chi = 2^q$ and the length of the sequence $|S|$.

Model species	$\lvert\theta\rvert$
uPTN	$D(\lvert\theta^l_w\rvert\lvert V\rvert + \lvert\theta^l_m\rvert + \lvert\theta^l_c\rvert)$
hPTN	$D(\lvert\theta^l_w\rvert\lvert V\rvert + \lvert\theta^l_m\rvert(\lvert S\rvert - 1) + \lvert\theta^l_c\rvert)$
uTTN	$D(\lvert\theta^l_w\rvert\lvert V\rvert + \lvert\theta^l_m\rvert + \lvert\theta^l_c\rvert)$
hTTN	$D(\lvert\theta^l_w\rvert\lvert V\rvert + \lvert\theta^l_m\rvert\log(\lvert S\rvert) + \lvert\theta^l_c\rvert)$
uSTN	$D(\lvert\theta^l_w\rvert\lvert V\rvert + \lvert\theta^l_m\rvert + \lvert\theta^l_c\rvert)$
rSTN	$\leq D(\lvert\theta^l_w\rvert\lvert V\rvert + \lvert\theta^l_m\rvert(\lvert S\rvert - 1) + \lvert\theta^l_c\rvert)$
hSTN	$\leq D(\lvert\theta^l_w\rvert\lvert V\rvert + \lvert\theta^l_m\rvert\lvert R\rvert + \lvert\theta^l_c\rvert)$
uCTN	$D(\lvert\theta^l_w\rvert\lvert V\rvert + \lvert\theta^l_m\rvert + \lvert\theta^l_f\rvert + \lvert\theta^l_c\rvert)$
hCTN	$D(\lvert\theta^l_w\rvert\lvert V\rvert + \lvert\theta^l_m\rvert\log(\lvert S\rvert) + \lvert\theta^l_f\rvert(\log(\lvert S\rvert) - 1) + \lvert\theta^l_c\rvert)$
uSCTN	$D(\lvert\theta^l_w\rvert\lvert V\rvert + \lvert\theta^l_m\rvert + \lvert\theta^l_f\rvert + \lvert\theta^l_c\rvert)$
rSCTN	$\leq D(\lvert\theta^l_w\rvert\lvert V\rvert + \lvert\theta^l_m\rvert\lvert R\rvert + \lvert\theta^l_f\rvert\lvert R\rvert + \lvert\theta^l_c\rvert)$
hSCTN	$\leq D(\lvert\theta^l_w\rvert\lvert V\rvert + \lvert\theta^l_m\rvert(\lvert S\rvert - 1) + \lvert\theta^l_f\rvert(\lvert S\rvert - 2) + \lvert\theta^l_c\rvert)$

Table 2: Number of parameters per model species.

2 Methods

2.1 Efficient Parameterised Quantum Circuits

The trainable tensors are given by circuit ansatz consisting of parameterized unitaries applied in parallel and sequence to qubits. Substitution of the parameters to the resulting symbolic functions can be very slow and inhibiting for large data training. However, we can get around this using JAX. Instead of constructing a symbolic function and substituting our updated parameters after each training iteration we can instead JAX compile a pure function that takes the parameter values as input and applies the numerical gate unitaries directly. This construction uses DisCoPy's quantum tensor network library. We then batch this operation using `jax.vmap` for further efficiency.

2.2 Batched contraction of PTN, TTN, STN

In order to run this model efficiently for large-scale data it is necessary to implement batching of the trees which, is non-trivial for the varying structures present in the syntactic model. We achieve this by padding the sentences to be of equal length, N, such that all trees have $N-1$ merge operations. We then define a length N vector for each tree where each element of this vector is either a classical vector, an initialised word state or a null pad state. When $\perp =$ `discard`, all quantum states are set to `mixed` such that density matrices and Kraus operators are used throughout. The syntax trees in our batch provide an ordering, a list of pairs of indices corresponding to the N-vectors, in which we need to apply the `merge` operations. We define a `merge` function that takes as input the relevant parameters defining this operation and the two elements taken from the N-vector according to the current tree indices. After the pooling-like operation we update the N-vector at the lower index with this result ready for it to be merged again later in the tree or measured. The pad indices are simply $(N-1, N)$ as the final classification state will always be at index 0 and hence updating the pad indices will not affect results. So the i-th `merge` operation can now be applied in parallel across all trees, again using `jax.vmap`, and we sweep over these operations sequentially until all $N-1$ are performed utilising `jax.lax.scan`. This allows for fast batched contractions which are easily run on GPUs for further efficiency. Discarding and measurement are all readily implemented using `DisCoPy`.

2.3 Batched contraction of CTN, SCTN

Due to the presence of disentanglers, the CTN calculation cannot be factorised as above so instead JIT compilation and `jax.vmap` is applied to the full contraction sequence. Additionally, the CTN must be padded to a length of 2^n, for integer n, so we pad to the minimum above the true length of the sequence. Batched contraction is then applied to this grouped data. For the SCTN, inability to factorise the contraction is significantly more limiting for simulation of these networks. Saving a JIT compiled model for all syntactic structures is too memory extensive. Thus, to present results for this model we select the 100 most frequently occurring syntactic structures present in the data and batch over the examples with shared structure.
