# Supplementary material for: Sequence processing with quantum-inspired tensor networks
Source: Sci Rep. 2025 Feb 28;15:7155. doi: 10.1038/s41598-024-84295-2 (PMC11871337; doi:10.1038/s41598-024-84295-2)
Supplement: Supplementary file 1 — Supplementary Information. [file 41598_2024_84295_MOESM1_ESM.zip › figures/h2.pdf]

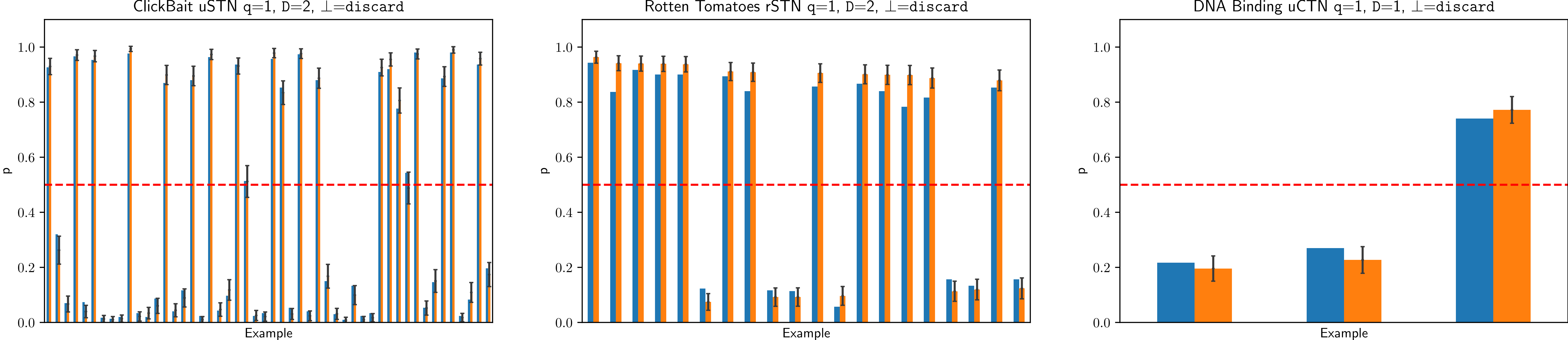

ClickBait uSTN q=1, D=2, ⊥=discard
Rotten Tomatoes rSTN q=1, D=2, ⊥=discard
DNA Binding uCTN q=1, D=1, ⊥=discard
p
Example
0.0
0.2
0.4
0.6
0.8
1.0
