# Supplementary figures and images for: Sequence processing with quantum-inspired tensor networks
Source: Sci Rep. 2025 Feb 28;15:7155. doi: 10.1038/s41598-024-84295-2 (PMC11871337; doi:10.1038/s41598-024-84295-2)

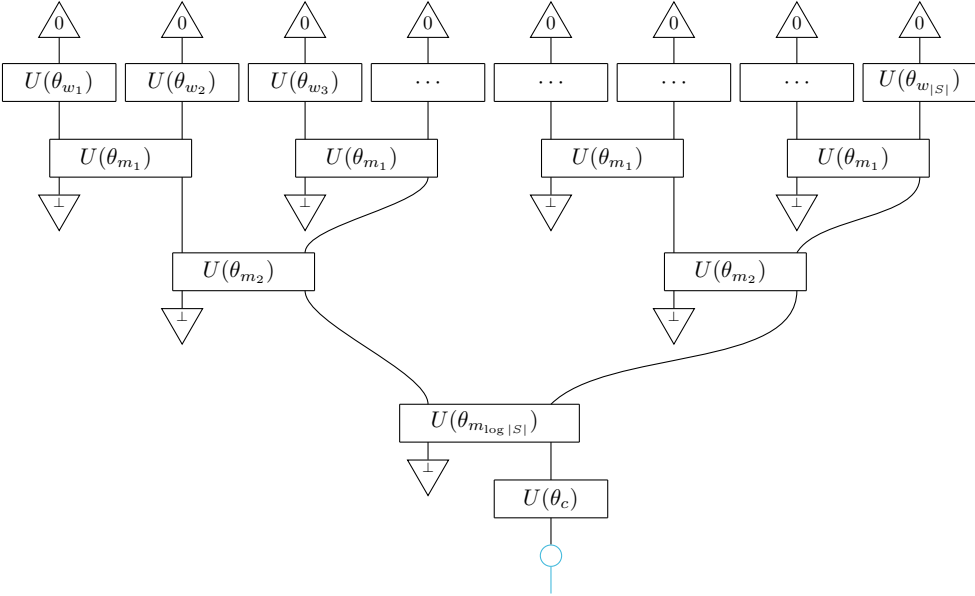

Supplement: Supplementary file 1 — Supplementary Information. [file 41598_2024_84295_MOESM1_ESM.zip › figures/tree-2.pdf]

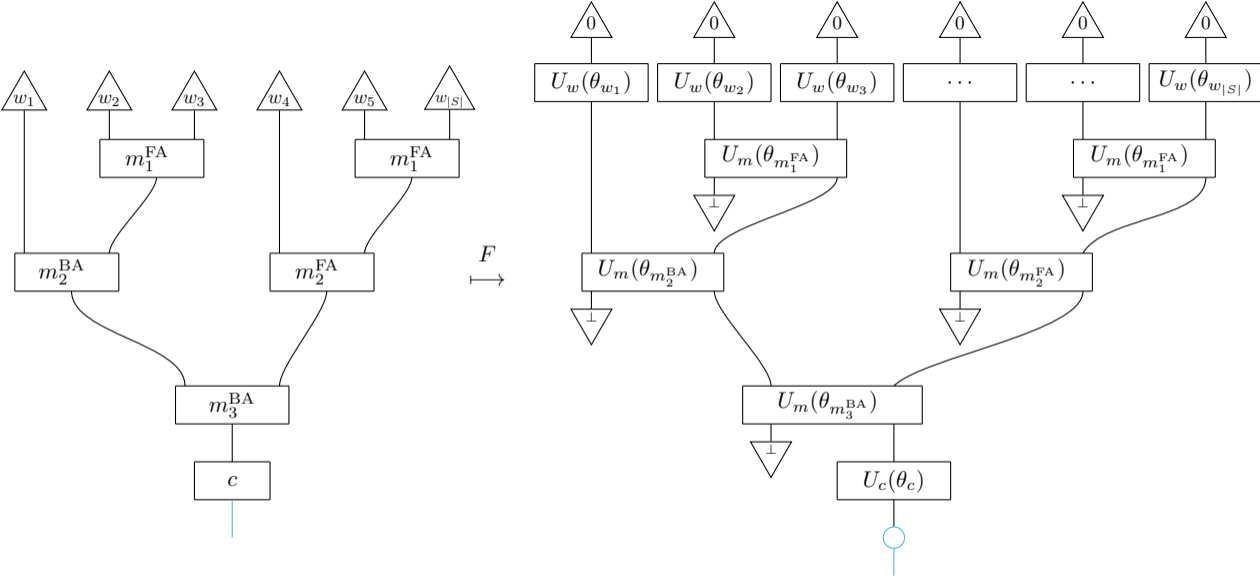

Supplement: Supplementary file 1 — Supplementary Information. [file 41598_2024_84295_MOESM1_ESM.zip › figures/syntax.pdf]

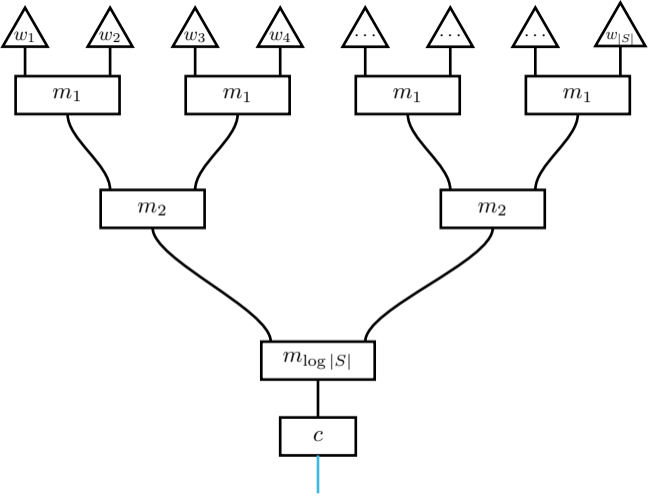

Supplement: Supplementary file 1 — Supplementary Information. [file 41598_2024_84295_MOESM1_ESM.zip › figures/tree-1.pdf]

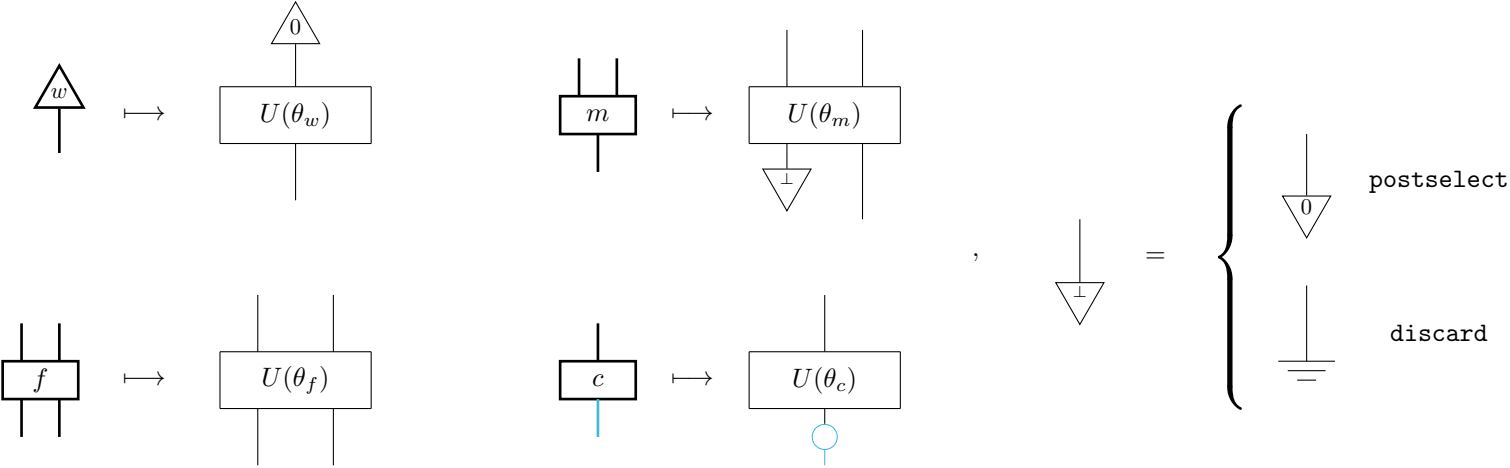

Supplement: Supplementary file 1 — Supplementary Information. [file 41598_2024_84295_MOESM1_ESM.zip › figures/functor.pdf]

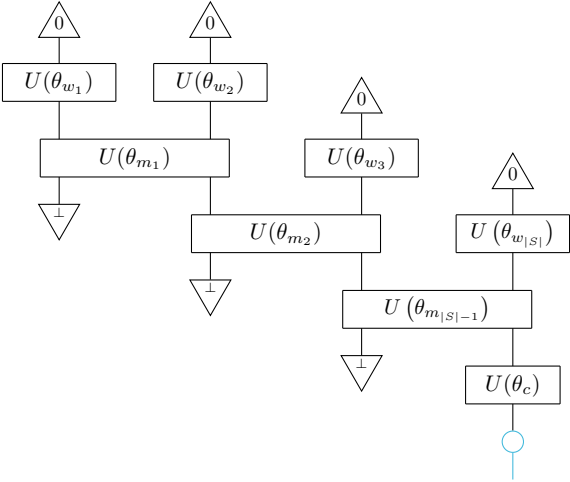

Supplement: Supplementary file 1 — Supplementary Information. [file 41598_2024_84295_MOESM1_ESM.zip › figures/path-2.pdf]

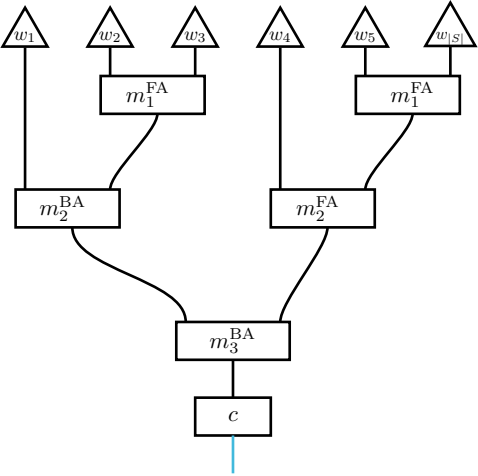

Supplement: Supplementary file 1 — Supplementary Information. [file 41598_2024_84295_MOESM1_ESM.zip › figures/syntax-1.pdf]

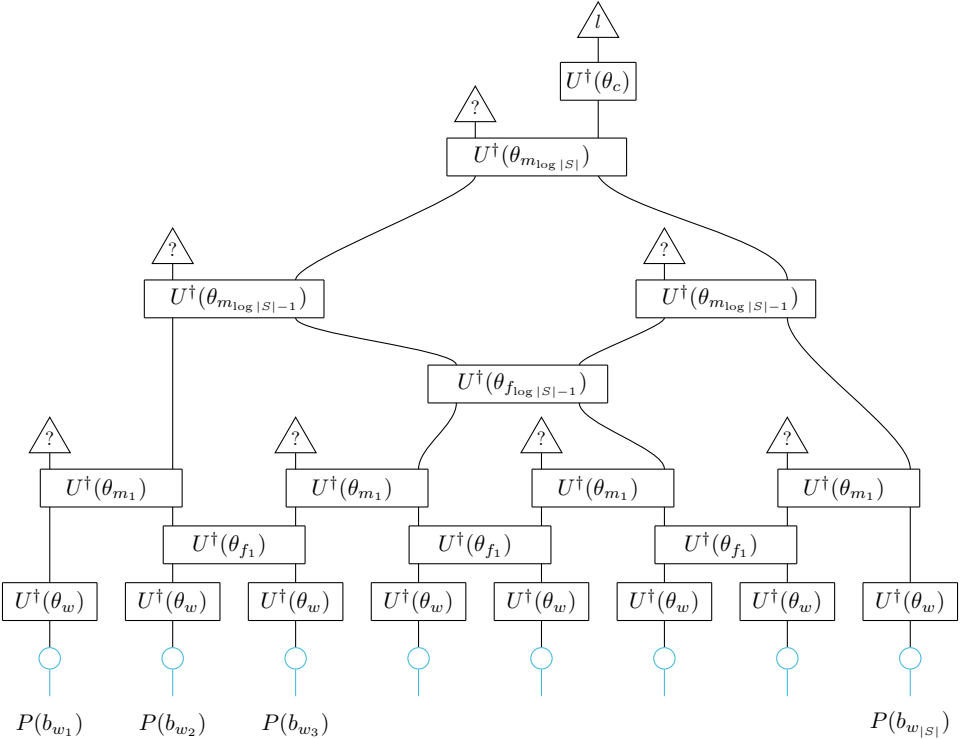

Supplement: Supplementary file 1 — Supplementary Information. [file 41598_2024_84295_MOESM1_ESM.zip › figures/gen-2.pdf]

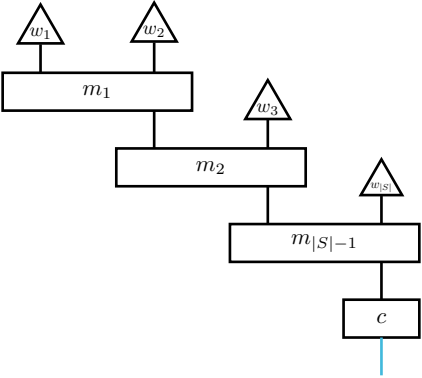

Supplement: Supplementary file 1 — Supplementary Information. [file 41598_2024_84295_MOESM1_ESM.zip › figures/path-1.pdf]

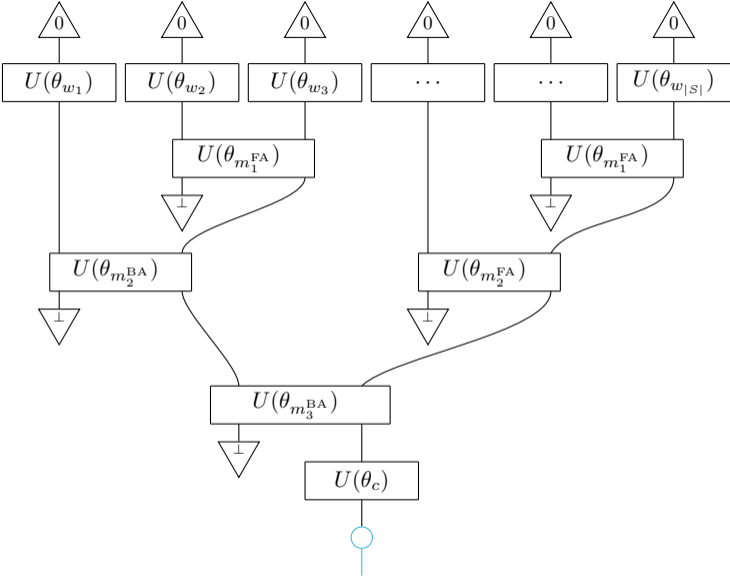

Supplement: Supplementary file 1 — Supplementary Information. [file 41598_2024_84295_MOESM1_ESM.zip › figures/syntax-2.pdf]

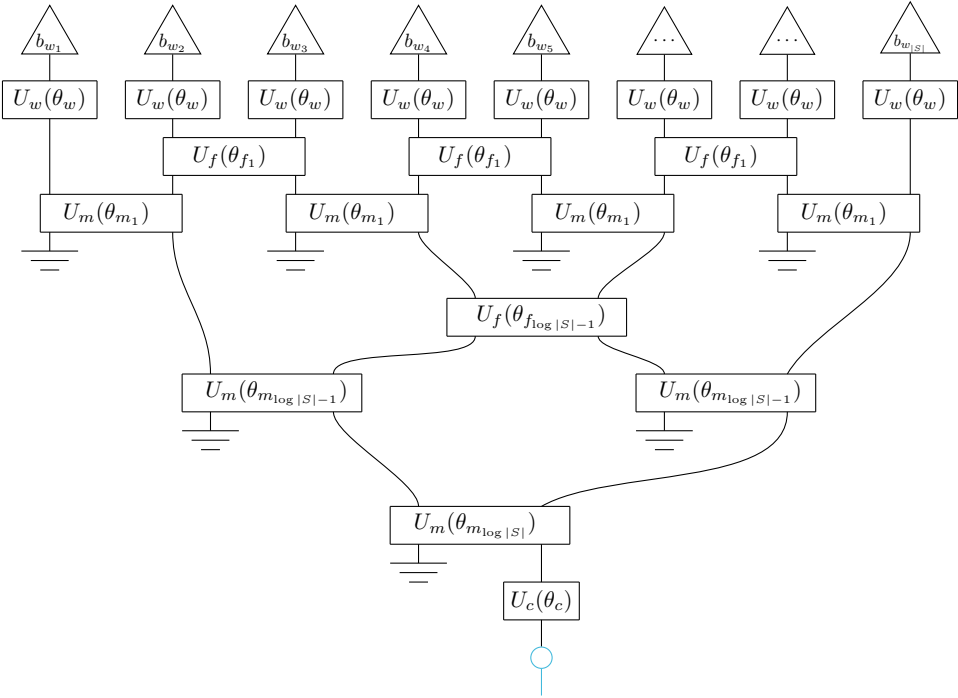

Supplement: Supplementary file 1 — Supplementary Information. [file 41598_2024_84295_MOESM1_ESM.zip › figures/gen-1.pdf]

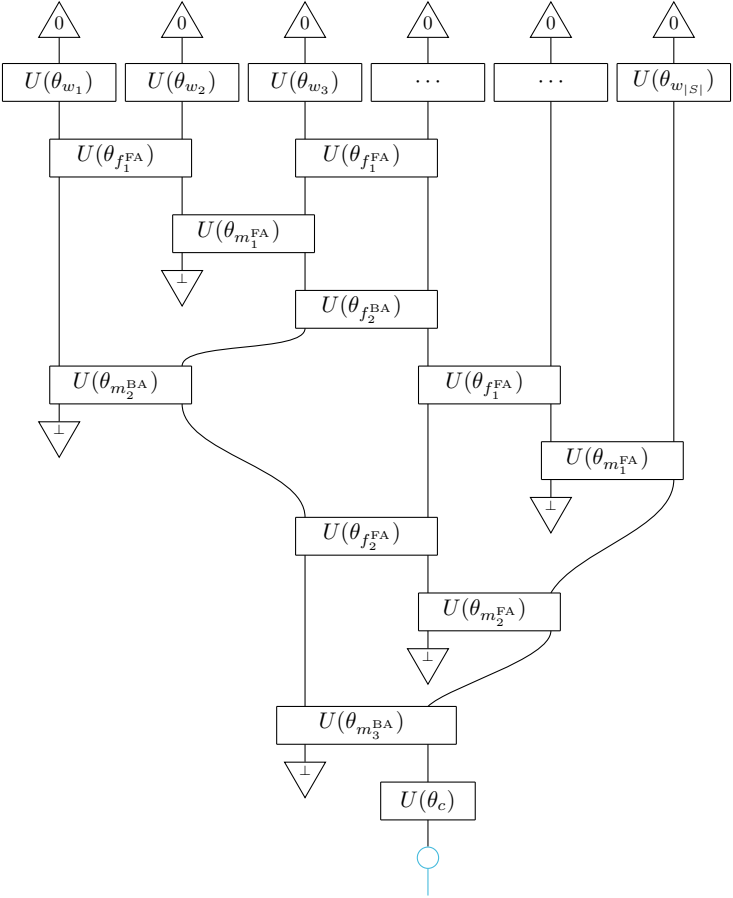

Supplement: Supplementary file 1 — Supplementary Information. [file 41598_2024_84295_MOESM1_ESM.zip › figures/syntax-conv-2.pdf]

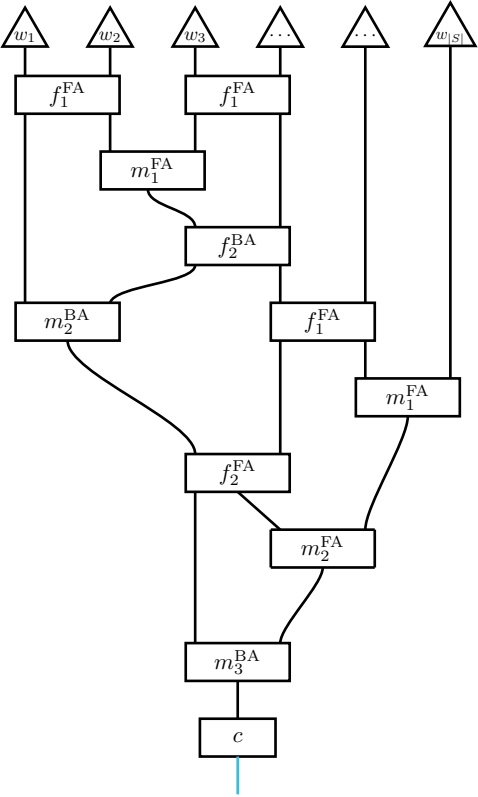

Supplement: Supplementary file 1 — Supplementary Information. [file 41598_2024_84295_MOESM1_ESM.zip › figures/syntax-conv-1.pdf]

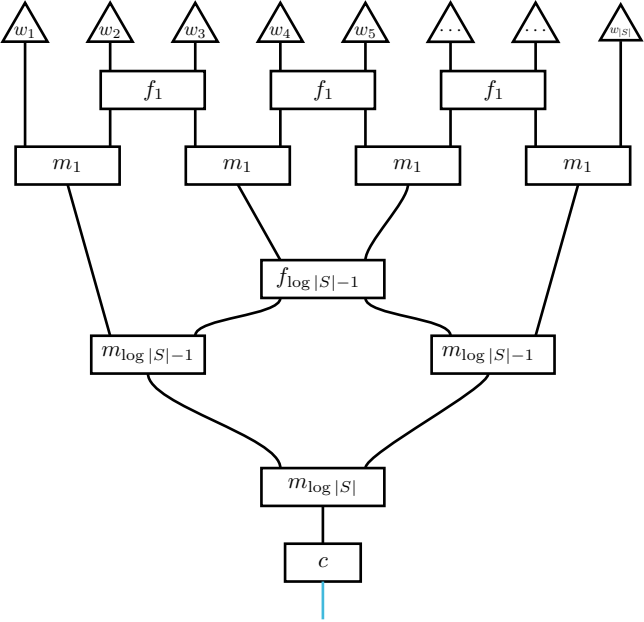

Supplement: Supplementary file 1 — Supplementary Information. [file 41598_2024_84295_MOESM1_ESM.zip › figures/conv-1.pdf]

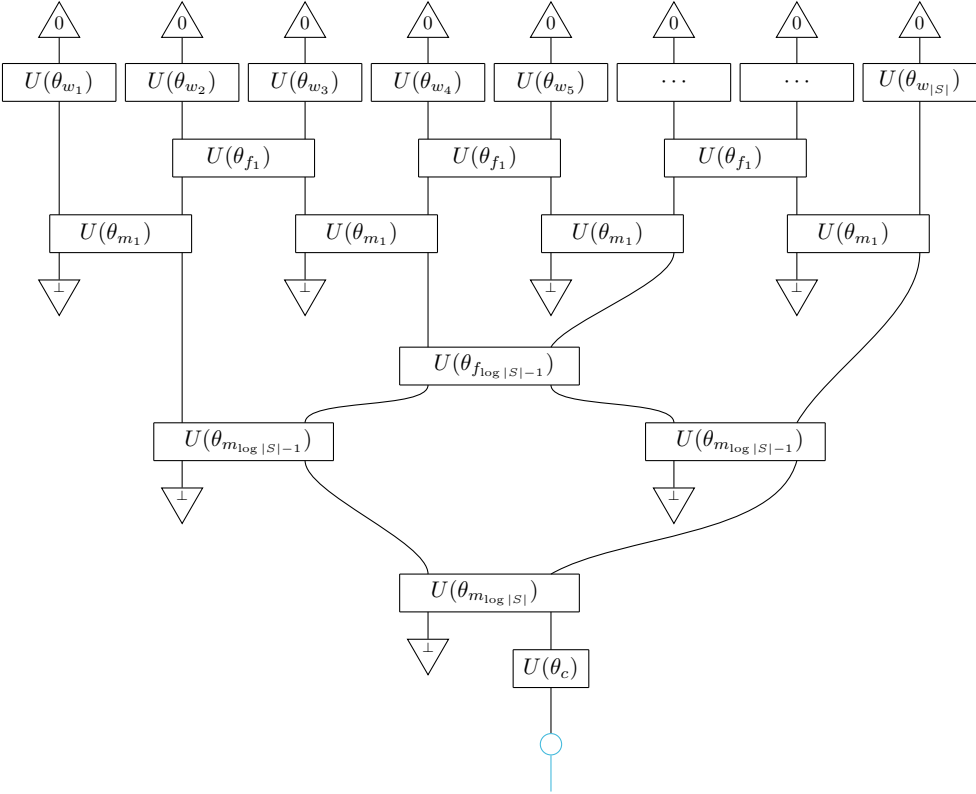

Supplement: Supplementary file 1 — Supplementary Information. [file 41598_2024_84295_MOESM1_ESM.zip › figures/conv-2.pdf]

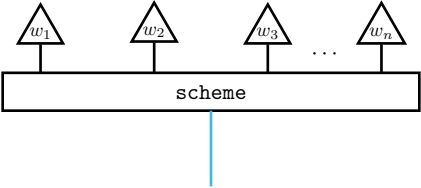

Supplement: Supplementary file 1 — Supplementary Information. [file 41598_2024_84295_MOESM1_ESM.zip › figures/scheme.pdf]

ClickBait uSTN  $q=1$ ,  $D=2$ ,  $\perp$ =discard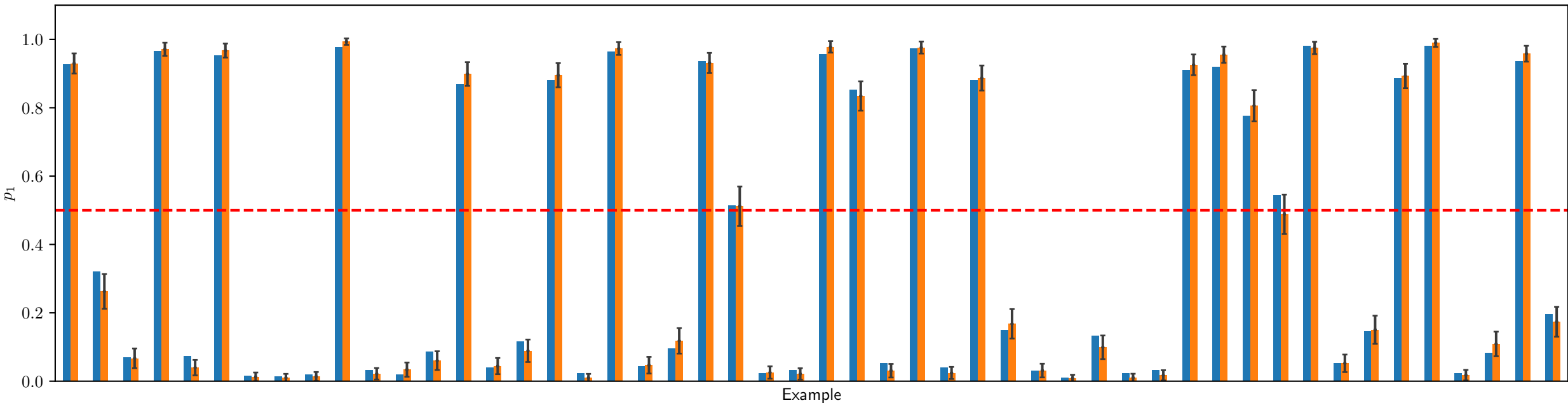

Supplement: Supplementary file 1 — Supplementary Information. [file 41598_2024_84295_MOESM1_ESM.zip › figures/h2-1.pdf]

Rotten Tomatoes rSTN  $q=1$ ,  $D=2$ ,  $\perp$ =discard

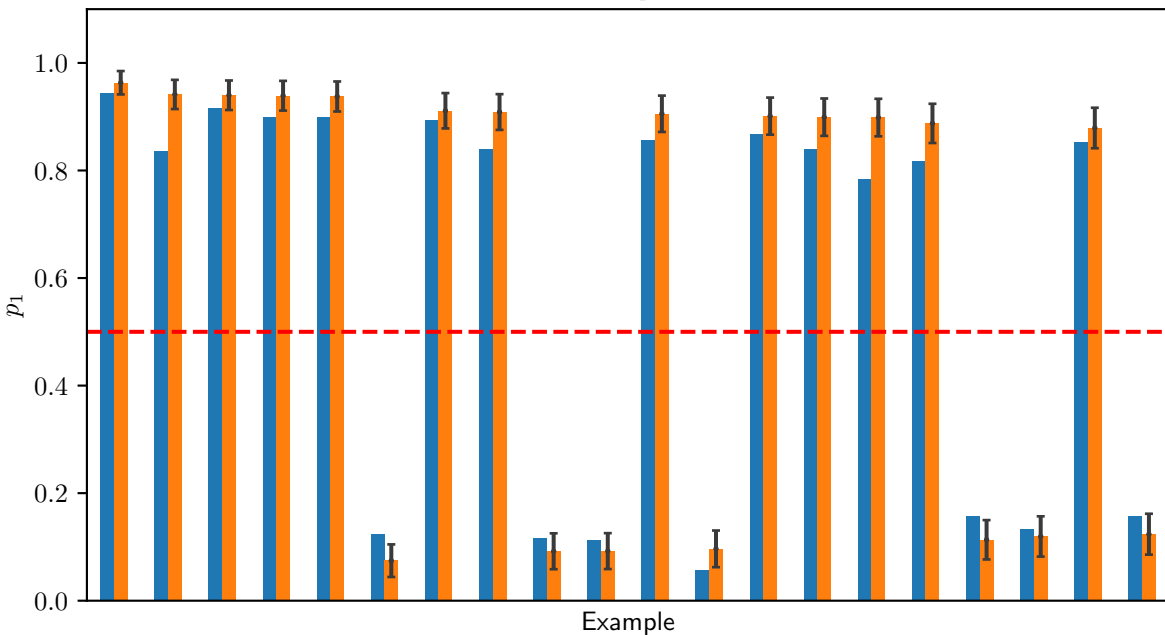

DNA Binding uCTN  $q=1$ ,  $D=1$ ,  $\perp$ =discard

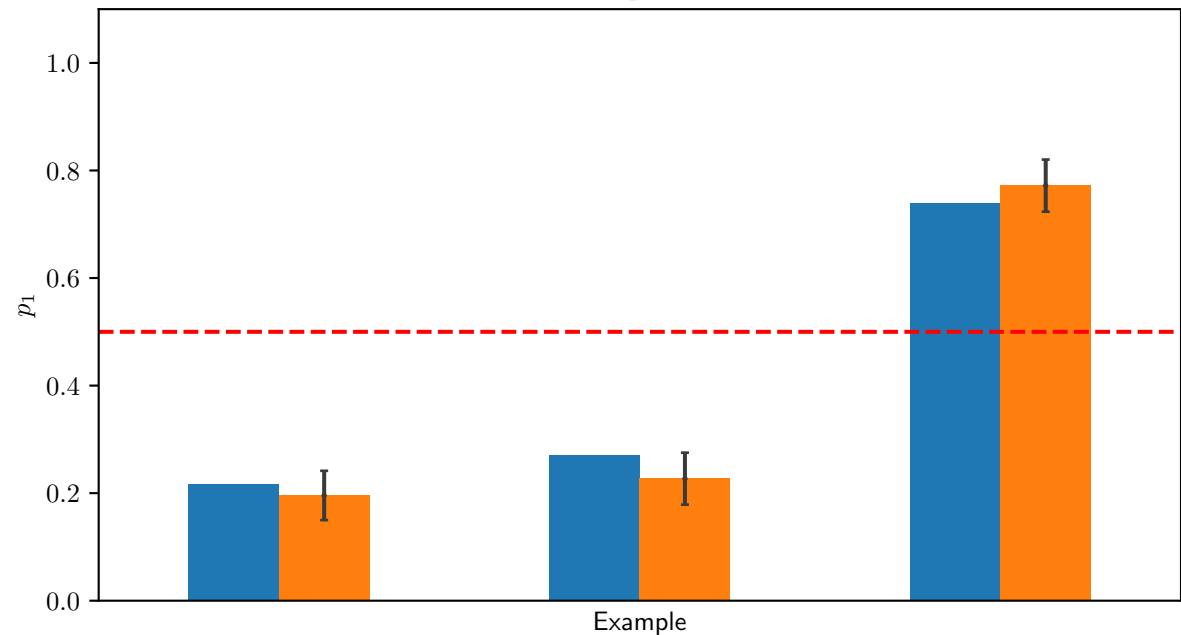

Supplement: Supplementary file 1 — Supplementary Information. [file 41598_2024_84295_MOESM1_ESM.zip › figures/h2-2.pdf]
